# Enhanced Thermoelectric Properties of P-Type Sn-Substituted Higher Manganese Silicides

**DOI:** 10.3390/nano14060494

**Published:** 2024-03-09

**Authors:** Ming-Xun Jiang, Sang-Ren Yang, I-Yu Tsao, Bayu Satriya Wardhana, Shih-Feng Hsueh, Jason Shian-Ching Jang, Cheng-Lun Hsin, Sheng-Wei Lee

**Affiliations:** 1 Institute of Materials Science and Engineering, National Central University, Taoyuan 32001, Taiwan; joshua35952@gmail.com (M.-X.J.); evauseonly@gmail.com (I.-Y.T.); jscjang@ncu.edu.tw (J.S.-C.J.); 2 Department of Electrical Engineering, National Central University, Taoyuan 32001, Taiwan; 3 Department of Mechanical Engineering, National Central University, Taoyuan 32001, Taiwan; 4 Department of Chemical and Materials Engineering, National Central University, Taoyuan 32001, Taiwan

**Keywords:** higher manganese silicide, thermoelectric, figure of merit, thermal conductivity, spark plasma sintering

## Abstract

This study introduces Sn-substituted higher manganese silicides (MnSi_1.75_, HMS) synthesized via an arc-melting process followed by spark plasma sintering (SPS). The influences of Sn concentrations on the thermoelectric performance of Mn(Si_1−x_Sn_x_)_1.75_ (x = 0, 0.001, 0.005, 0.01, 0.015) are systematically investigated. Our findings reveal that metallic Sn precipitates within the Mn(Si_1−x_Sn_x_)_1.75_ matrix at x ≥ 0.005, with a determined solubility limit of approximately x = 0.001. In addition, substituting Si with Sn effectively reduces the lattice thermal conductivity of HMS by introducing point defect scattering. In contrast to the undoped HMS, the lattice thermal conductivity decreases to a minimum value of 2.0 W/mK at 750 K for the Mn(Si_0.999_Sn_0.001_)_1.75_ sample, marking a substantial 47.4% reduction. Consequently, a figure of merit (ZT) value of ~0.31 is attained at 750 K. This considerable enhancement in ZT is primarily attributed to the suppressed lattice thermal conductivity resulting from Sn substitution.

## 1. Introduction

Thermoelectric (TE) materials, known for their ability to convert heat energy directly into electricity and vice versa, hold significant promise in waste heat recovery and refrigeration applications. The main challenge of the TE technology relies on developing TE materials with higher figure of merit (*ZT*) in order to improve efficiencies of TE devices. The performance of TE materials hinges on a dimensionless ZT *= S*^2^*σT*/*k_t_*, which amalgamates contributions from the Seebeck coefficient (*S*), electrical conductivity (*σ*), total thermal conductivity (*k_total_*), and absolute temperature (*T*) [1]. Recent advancements have demonstrated high-performance materials exhibiting peak ZT values across low, intermediate, and high temperature ranges within bismuth antimony telluride [2], silicides [3], and Zintl phase materials [4], respectively. Among the silicides, manganese silicides have received particular attention due to their unique physical properties. There are seven thermodynamically stable phases in this family, namely MnSi_1.7_ (tetragonal), MnSi (cubic), Mn_5_Si_3_ (hexagonal), Mn_5_Si_2_ (tetragonal), Mn_3_Si (cubic), Mn_4_Si (rhombohedral), and Mn_6_Si (rhombohedral) [5]. In terms of magnetic properties, MnSi has a weak ferromagnetic property at a Curie temperature (*T_c_*) of 30 K. In the family of higher manganese silicides (HMS), the bulk state of Mn_4_Si_7_ has a very low FM or no magnetic moment in the bulk state, while the phases of Mn_11_Si_19_ and Mn_15_Si_26_ have small magnetic moments.

Higher manganese silicides, Si-rich compounds with a Si/Mn ratio ranging from 1.71 to 1.75 [6], stand out due to their non-toxic, environmentally friendly nature, high oxidation resistance, and abundance in the earth, positioning them as compelling p-type TE candidates. These materials exhibit robust chemical stability within the 500–800 K temperature range [7]. The cost of HMS is US$1.50 per kilogram. It has its advantages compared to other high-performance but expensive materials, for example, Bi_2_Te_3_ (US$ 110/kg) [8]. HMS, characterized by the alteration of transport properties and bonding characteristics via the d-orbital electrons of manganese, yield a higher Seebeck coefficient (ranging between 150 and 200 µV/K) [9]. Their crystal structure comprises interpenetrating sub-lattices forming the Nowotny chimney ladder (NCL) phases. This structure involves helical Si sub-lattices (space group *P4/nnc*) forming a ladder structure along the c-axis within the chimney-like tetragonal Mn sub-lattices (space group *I41/amd*) [10,11,12,13]. The crystal structures of HMS display tetragonal symmetry, with nearly identical lattice parameters around 5.5 Å. Crystal structure analysis has identified four HMS compositions exhibiting commensurate Nowotny structures, with c-axis lengths ranging from 17 to 118 Å: Mn_4_Si_7_, Mn_11_Si_19_, Mn_15_Si_26_, and Mn_27_Si_47_ [14,15]. However, the formation of HMS unavoidably accompanies the generation of cubic MnSi due to peritectic reactions and the slower diffusion rate of Si atoms. The low electrical and high thermal conductivity of MnSi may impede the TE performance of HMS [16].

Previous research has explored various strategies to enhance the ZT value of TE materials. These approaches encompass reducing lattice thermal conductivity through nanostructuring [17,18], amplifying alloy scattering [19], optimizing the power factor (*PF = S²σ*) by adjusting the carrier concentration [20], and boosting the Seebeck coefficient through band engineering [21]. Studies focusing on HMS materials have emphasized curbing the lattice thermal conductivity by integrating nanoparticles [22] and refining the electrical transport properties through elemental substitution [23,24]. Substituting Ge for Si in HMS notably decreased lattice thermal conductivity, yielding a ZT value of up to 0.6 at 833 K [25,26]. This reduction in lattice thermal conductivity was attributed to the introduction of defects into the lattice structure, substantially increasing phonon scattering. Introducing Al also led to defects in the crystal structure and increased charge carrier concentration, resulting in a slightly elevated ZT of 0.65 at 800 K [27]. However, substituting Si with Re did not affect the power factor significantly; instead, it primarily reduced thermal conductivity, resulting in a ZT of 0.57 at 920 K [28,29].

The introduction of heavier atoms in HMS, such as Sn instead of Si, can provoke mass and strain fluctuations within the material. These fluctuations play a crucial role in intensifying alloy scattering, thus leading to a reduction in lattice thermal conductivity [29]. Moreover, the larger atomic radius of Sn also enhances phonon scattering and thermoelectric effects in HMS.

Therefore, Sn-substituted HMS, specifically Mn(Si_1−x_Sn_x_)_1.75_ (x = 0, 0.001, 0.005, 0.010, 0.015), were synthesized using vacuum arc-melting followed by spark plasma sintering (SPS) in this work. The influences of Sn-substitution in HMS on both structural and thermoelectric properties were systematically studied.

## 2. Materials and Methods

### 2.1. Synthesis of Bulk HMS

**Figure 1:**
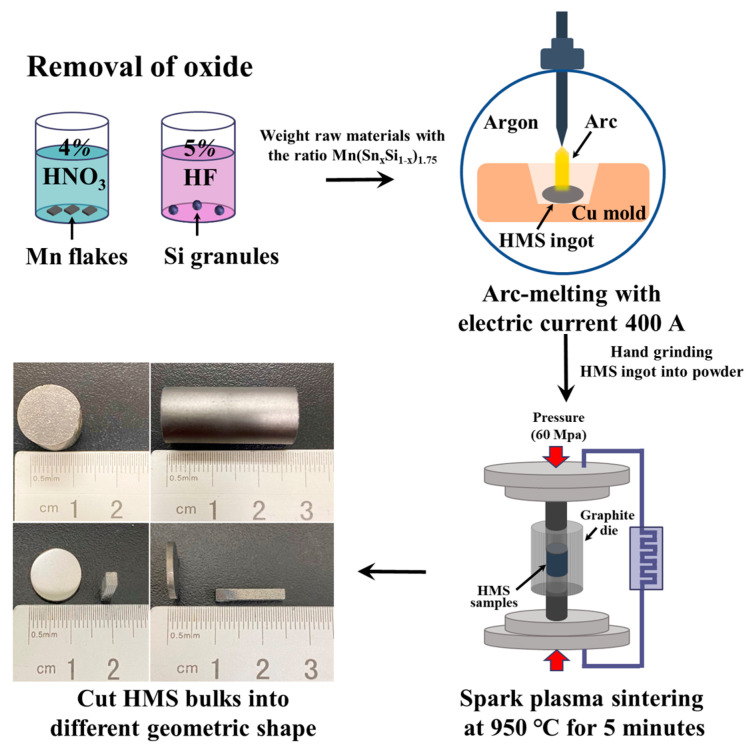
Fabrication process flowchart for Sn-Substituted HMS ingot.

Mn flakes (Alfa Aesar, 99.98%), Si powders (Summit-tech, 99.999%), and Sn powders (Summit-tech, 99.999%) were precisely weighed to attain the nominal atomic ratios required for Mn(Si_1−x_Sn_x_)_1.75_ (x = 0, 0.001, 0.005, 0.010, 0.015). To ensure accurate measurements, the oxide layer on Mn flakes was eliminated by washing it with 4% nitric acid, while the Si powders underwent a similar process using 5% hydrofluoric acid. Operating within an argon atmosphere for protection, the blended raw materials were arc-melted within a copper mold, employing an electric current of 400 A. Initially, a Ti rod was arc melted to absorb the residual oxygen within the cavity. To guarantee uniformity, the HMS ingot underwent multiple arc-melting cycles, being flipped at least three times during this process. During the process, the copper mold was continuously cooled with water. Subsequently, the oxide layer on the surface of the resultant ingots was removed using sandpaper and subsequently ground into a fine powder (325 mesh), which was then directly loaded into graphite dies for spark plasma sintering (SPS-515S, Fuji Electronic Industrial Co., Ltd., Tokyo, Japan). To improve the conductivity and facilitate the sintering process, carbon paper was positioned between the powder and the mold. This sintering process occurred under vacuum at 60 MPa and 950 °C for 5 min. Subsequently, the carbon paper adhered to the specimens’ surfaces was removed. The ingots were trimmed to the specified dimensions in accordance with the measuring equipment’s specifications. LFA samples were shaped into 10 × 10 mm squares with a thickness ranging from 1 to 3 mm, while Zem3 samples were cut into squares of 2 to 4 mm with lengths of 14 to 18 mm. For clarity, the fabricated material and the procedural steps are illustrated in Figure 1.

### 2.2. Material Characterizations

The sintered Mn(Si_1−x_Sn_x_)_1.75_ samples were examined under an x-ray diffractometer (XRD, Bruker D2 phaser) with Cu Kα radiation, *λ* = 0.154 nm for phase identification. The surface morphology of the sintered samples was characterized by using field-emission scanning electron microscopy (FESEM, FEI Quanta 200F) in the backscattered electron (BSE) mode. For high-resolution transmission electron microscopy (HRTEM), bulk HMS samples were precisely sliced and mounted onto carbon film grids using focused ion beam (FIB) technology. The JEOL JEM-2100 microscope, operating at an accelerating voltage of 200 kV, enabled detailed analysis. The density of the sintered samples was determined using the Archimedes method, employing water as the liquid medium for measurement.

### 2.3. Measurements of Thermoelectric Properties 

The electrical conductivity and Seebeck coefficient (*S =* ∆*V*/∆*T*) were concurrently measured using the standard four-probe method (Sinkuriko: ZEM-3) in an argon atmosphere. The thermal diffusivity (*D*) and specific heat capacity (*C_p_*) were determined employing a laser flash method (Netzsch: LFA 457) and a power-compensation differential scanning calorimeter (Netzsch, STA 449 F3) in a vacuum, respectively. All measurements were conducted within the temperature range of 323 K to 748 K. Thermal conductivity can be derived from the measured density, specific heat capacity, and thermal diffusivity using the relationship *k_total_ = d C_p_ D*. Furthermore, the room-temperature Hall coefficient (*R_H_*) was obtained by applying a current of approximately 6 mA, followed by a magnetic field change of 1.6 T. Subsequently, the carrier concentration (*n*) and Hall mobility (*μ_H_*) were computed as *n =* 1/(*e R_H_*) and *μ = σ R_H_*, respectively, where ‘*e*’ represents the electronic charge.

## 3. Results and Discussion

### 3.1. Material Characterizations of Sn-Substituted HMS

**Figure 2:**
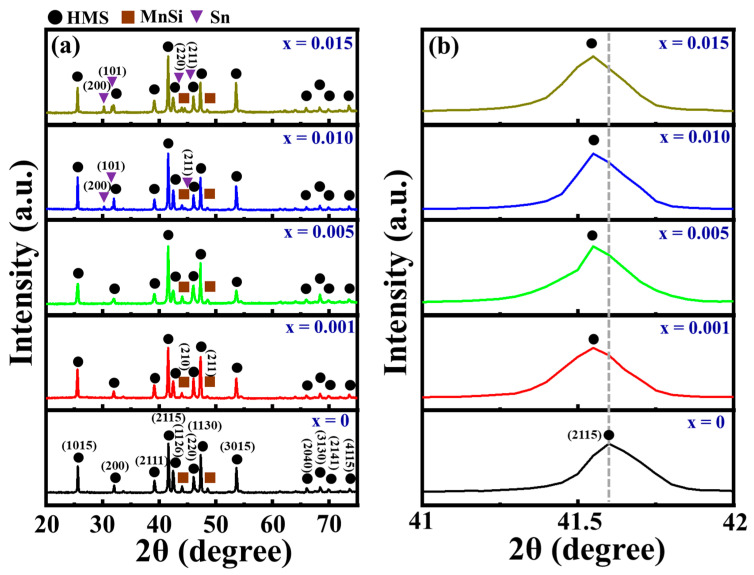
(**a**) XRD pattern of the sintered Mn(Si_1−x_Sn_x_)_1.75_ samples with different Sn doping amounts and (**b**) the partial enlarged view around 42° in (**a**).

Figure 2a shows the X-ray diffraction (XRD) patterns of the sintered Sn-substituted HMS bulk samples. It was observed that all samples exhibited characteristic peaks of HMS materials (reference JCPDS # 89-2413) alongside diffraction peaks indicating the presence of a secondary phase, MnSi. The MnSi phase is known to potentially diminish TE properties due to its narrow band gap and high electrical conductivity [30]. Referring to the Mn-Si binary phase diagram [31], HMS gradually forms during the arc-melting process when the molten mixture reaches the liquidus stage. Owing to the sluggish diffusion rate of Si atoms, the MnSi phase typically coexists within the HMS matrix. Concurrently, the metallic Sn peak becomes evident at x = 0.005. The increase in carrier concentration from Sn is expected to result in a decrease in the Seebeck coefficient.

Figure 2b further presents the XRD patterns around 40° for the sintered Sn-doped HMS samples. Gradual shifts of peaks towards lower angles were observed with increased Sn content, signifying an expansion in HMS unit cells and lattice parameters due to Sn substitution. The obtained HMS powders typically contain multiple phases with varied *c* lattice parameters, resulting in slight deviations compared to the single HMS phase. In this study, all lattice parameters of the HMS samples were computed from XRD peak positions with the equation: *Sin*^2^*θ* = *λ*^2^/(4*a*)^2^ (*h*^2^ + *K*^2^) + *λ*^2^/(4*c*)^2^(*l*^2^). The findings revealed that both the average lattice parameters a and c of Mn(Si_1−x_Sn_x_)_1.75_ (x = 0.001) were higher than those of the undoped HMS. This increase is attributed to substituting the smaller atomic radius of Si (*r_Si_* = 1.11 Å) with the larger Sn (*r_Sn_* = 1.40 Å). However, as Sn content surpassed 0.1 at%, the mean lattice parameters remained constant. The lattice parameters of HMS are shown in Table 1. This suggests that the solubility limit of Sn in Sn-substituted HMS bulk samples is approximately at x = 0.001.

**Figure 3:**
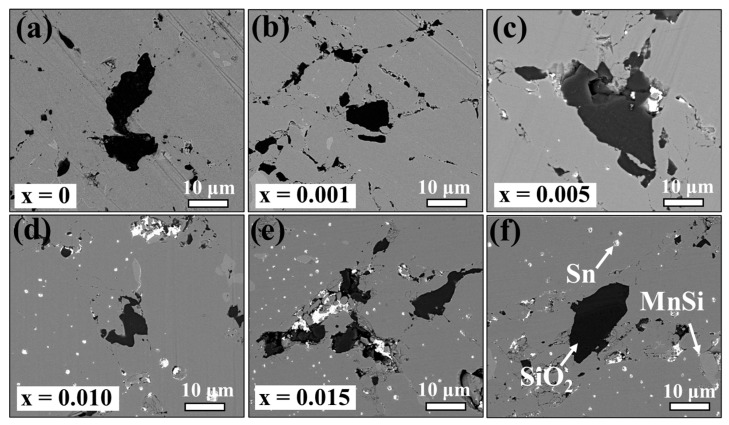
BSE images showing surface morphology of the sintered Mn(Si_1−x_Sn_x_)_1.75_ pellets with different Sn doping amounts: (**a**) x = 0, (**b**) x = 0.001, (**c**) x = 0.005, (**d**) x = 0.010, (**e**), and (f) x = 0.015. Distinct contrasts within the images denote various components: HMS, Si, Sn, and MnSi, indicated respectively.

**Figure 4:**
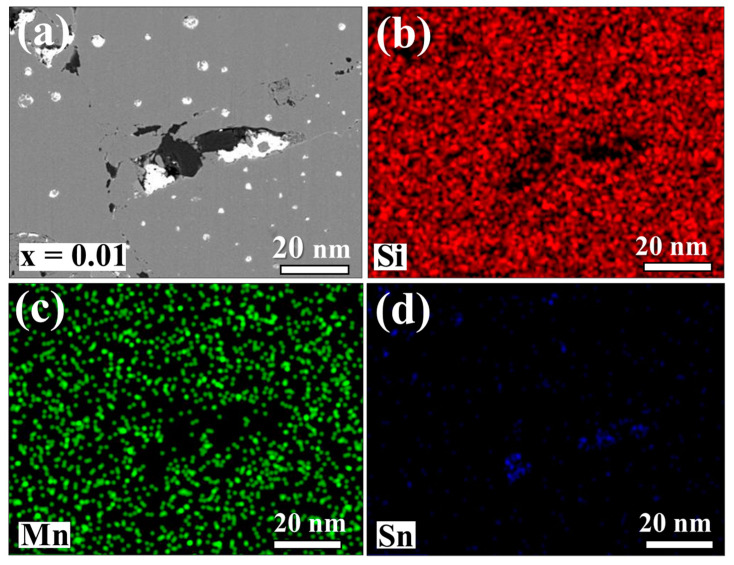
EDS mapping images of the sintered Mn(Si_0.99_Sn_0.01_)_1.75_ sample: (**a**) the selected area BSE image; (**b**–**d**) represent the signals corresponding to Si, Mn, and Sn, respectively.

**Figure 5:**
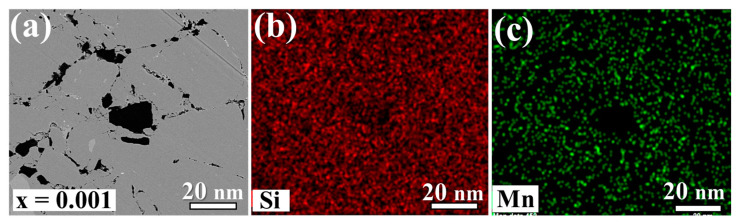
EDS mapping images of the sintered Mn(Si_0.999_Sn_0.001_)_1.75_ sample: (**a**) the selected area BSE image; (**b**,**c**) represent the signals corresponding to Si, and Mn, respectively.

Figure 3 displays BSE images revealing an inhomogeneous micromorphology on the surfaces of Sn-substituted HMS bulk materials. The color contrast highlights distinct phases attributed to variations in atomic order, with brighter regions indicating higher atomic order. Within the light grey areas lies the MnSi phase, typically inherent within the HMS matrix, which is consistent with the EDS mapping results depicted in Figure 4 and Figure 5. As the Sn content increased up to 0.5 at%, discernible bright regions representing the metallic Sn phase appears, suggesting the precipitation of Sn within the HMS matrix. This implies a solubility limit of approximately 0.1 at% in Mn(Si_1−x_Sn_x_)_1.75_. These findings are consistent with the observations from the XRD results. It is our conjecture that the residual Si underwent significant oxidation during the synthesis process. As a result, the residual Si is detectable in the BSE images. However, there are no discernible peaks attributable to Si, likely due to the formation of amorphous SiO_2_.

**Figure 6:**
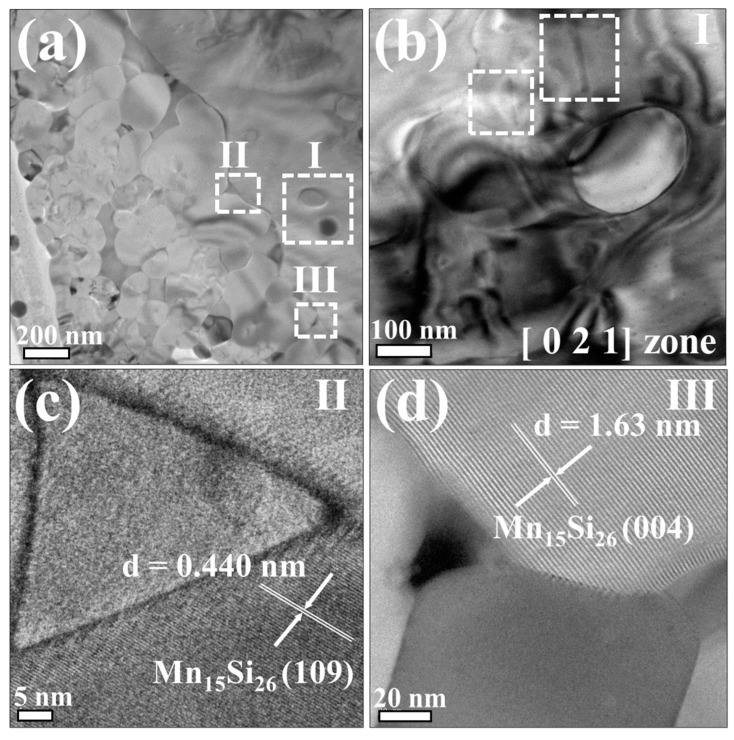
TEM images of the sintered Mn(Si_0.985_Sn_0.015_)_1.75_ sample: (**a**) low-magnification image illustrating the grain size of the sintered sample; (**b**) highlighting the strain field and stacking fault around the precipitated Sn particles in the region (I) of (**a**); (**c**,**d**) showing the high-resolution lattice images capturing different crystal planes in the region (II) and (III).

Previous literature based on first-principle calculations has reported the significant influence of atomic arrangement and defects on the band structure of HMS [32]. In Figure 5a, a typical low-magnification TEM image of the sintered Mn(Si_0_._985_Sn_0.015_)_1.75_ sample is presented, revealing a grain size ranging from approximately 100 nm to 450 nm. This grain size distribution is anticipated to effectively scatter long-wavelength phonons. Three distinct regions (I), (II), and (III) marked in Figure 6a were selected for dislocation and lattice plane analysis. The magnified images are shown in Figure 6b, c, and d respectively. The diffraction contrast observed in these regions is a result of altering the incident beam tilt on the Bragg planes of crystal structure, revealing dislocations or defects. Figure 6b indicates the presence of stacking faults, alongside dislocations possibly induced by the inclusion of Sn within the HMS matrix. Figure 6c,d further show the lattice images of regions II and III, revealing crystal structures corresponding to Mn_15_Si_26_.

**Figure 7:**
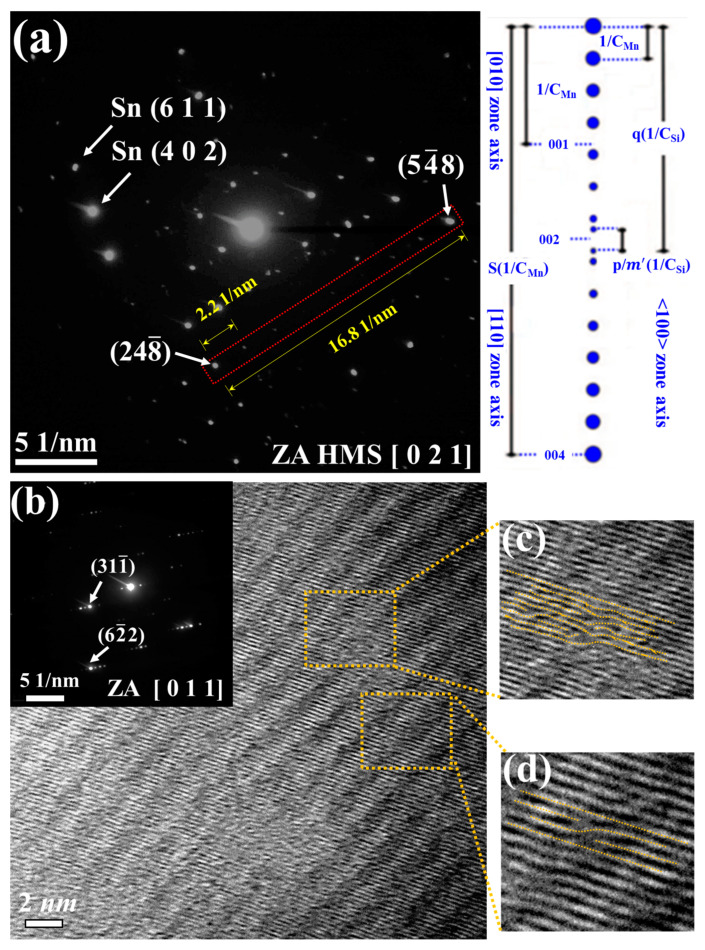
(**a**) SAED image along the zone axis [021]. (**b**) TEM image showing fringes resulting from the interference of superposed crystals. (**c**) Enlarged specific area revealing a stacking fault within the Si fringes and (**d**) showing a deficient half fringe.

The HMS phases were also identified by selected area diffraction (SAED) along lower index zone axes [1 0 0], [1 1 0], and [1 2 0], primarily differing in their c parameters [33,34]. In Figure 7a, the SAED pattern along the [021] zone axis reveals distinct diffraction spot arrays, which can be identified as Sn and HMS, respectively. In addition, the most intense main spots within the HMS are contributed from the centered tetragonal sub-lattices of Mn atoms. There are linear satellite spots sequence associated with reciprocal Si sub-lattice spacing between two main spots. The equation that expresses the relation between main spots and satellite spots is (2q − p/m’) (1/C_Si_) = s(1/C_Mn_) [34]. The integer q and s is dependent on the zone axis of SAED. Along the zone axis [1 2 0], the value of the integer is q = 4 and s = 2, respectively. The meaning of p/m’ (1/C_Si_) is the distance of the mismatch region in the satellite spots sequence center. The determination of the HMS phase becomes feasible by integrating it with another relationship, C = nC_Mn_ = mC_Si_, where m = m’ and n = [2(q + 1)m − p]/4 [34]. The HMS phase can be identified specifically as Mn_15_Si_26_.

In Figure 7b, two distinct fringes are also observed, resulting from the interference of superposed crystals. One comprises closer spaced fringes corresponding to the Si sub-lattices, while the other comprises wider spaced Moiré fringes intersecting the Si fringes at an angle. Notably, Figure 7c further reveals the presence of a stacking fault within the Si fringes, manifesting as a single lattice plane inserted into the periodic structure, potentially accompanied by distortions induced by the accumulation of point defects. The influences of these defects on TE properties will be discussed later. In addition, Figure 7d exhibits dislocation-like fringes. This pattern shows a deficient half fringe, possibly resulting from a helical arrangement of Si atoms shifted relative to each other along the c-direction.

### 3.2. TE Properties of Sn-Substituted HMS

**Figure 8:**
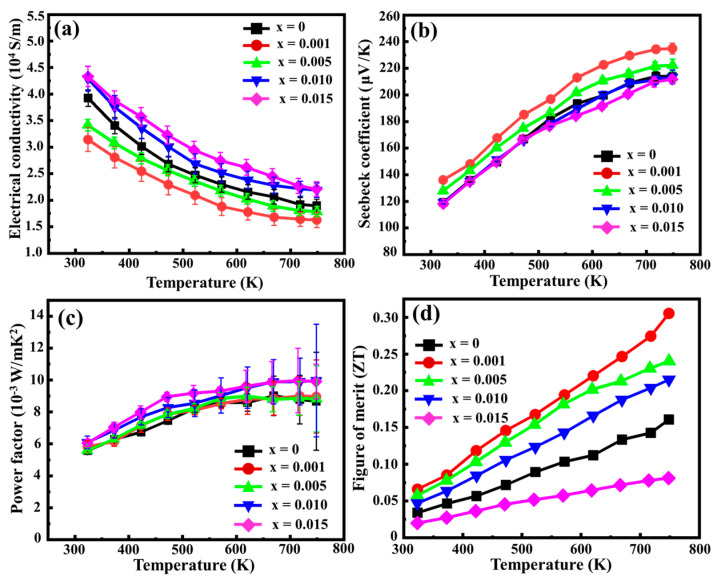
TE properties: (**a**) electrical conductivity, (**b**) Seebeck coefficient, (**c**) power factor, and (**d**) ZT values as a function of temperature for the Mn(Si_1−x_Sn_x_)_1.75_ samples.

Room-temperature Hall measurements were conducted to determine the room-temperature hole concentration (*ρ*) and mobility (*μ*), as summarized in Table 2. Quantifying the exact composition of each phase posed a challenge. Therefore, the Reference Intensity Ratio (RIR) method was employed to roughly determine the phase compositions [34]. The percentages of these phases are also presented in Table 2. The ratios of HMS to MnSi remain almost the same across all Sn-substituted samples. Furthermore, the observed precipitation of Sn when x ≥ 0.01 is also consistent with the SEM observations. The positive Hall coefficients observed in Sn-substituted HMS indicate that all of them are p-type TE materials. Previous studies have reported that iso-electronic substitution may disturb the ordering arrangement of Si and Mn atoms, potentially leading to alterations in the density of states (DOS) near the Fermi level. This alteration could arise from the possible formation of stacking faults within the HMS [14,35]. As a result, Sn substitution is likely to induce changes in the electronic band structure and carrier density in Mn(Si_1−x_Sn_x_)_1.75_. This inference is supported by the fact of a slight increase in hole concentration in HMS due to Sn substitution. However, the hole mobility of HMS decreases as the Sn doping amount increases, primarily due to the effects of alloy scattering on the carrier transport. The electrical conductivities (*σ*) of the Sn-substituted HMS samples are shown in Figure 8a. All HMS samples exhibit the characteristics of degenerate semiconductors, having a metal-like temperature dependence of σ below 800 K. The electrical conductivities in Mn(Si_1−x_Sn_x_)_1.75_ decrease from x = 0 to x = 0.001 due to a reduction in hole mobility. However, with further increasing the amounts of Sn doping from x = 0.001 to x = 0.015, the electrical conductivities increase significantly, potentially attributed to an increase in hole concentration and the formation of metallic Sn precipitates.

Normally, the Seebeck coefficients show opposite trend with the electrical conductivities, as shown in Figure 8b. The Seebeck coefficients observed in Mn(Si_1−x_Sn_x_)_1.75_ exhibited an increase trend with rising temperatures, consistent with the trends observed in the Hall coefficients. The temperature dependence of the Seebeck coefficient reveals a distinct plateau, marking the initiation of intrinsic electron excitations around 750 K. Utilizing the Goldsmid–Sharp band gap formula *E_g_ =* 2*e*|*S_max_|T_max_*, where *T_max_* represents the temperature corresponding to the maximum Seebeck coefficient (*S_max_*) [36], the band gap of Mn(Si_1−x_Sn_x_)_1.75_ can be approximately calculated. In this study, the calculated band gaps for the samples synthesized via the SPS process ranged between 0.32 and 0.34 eV. Furthermore, the temperature dependence of the power factor (PF) in Sn-substituted HMS is shown in Figure 8c. The power factors observed in the Sn-substituted HMS samples exhibited slightly higher values compared to the undoped counterparts. The highest power factor reaches 9.98 × 10^−4^ mW/mK², indicating a remarkable 13.6% increase relative to the undoped sample, achieved by the 1.5 at% Sn-substituted sample at 700 K.

**Figure 9:**
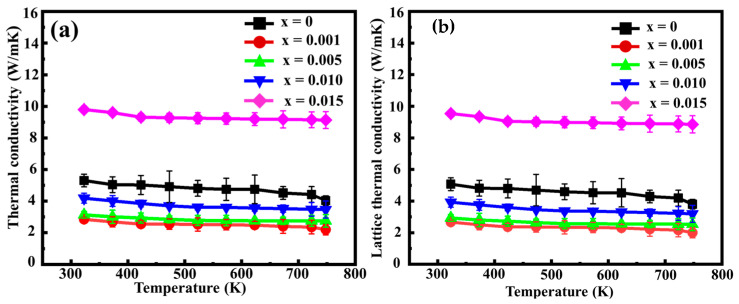
(**a**) The total thermal conductivity and (**b**) lattice thermal conductivity as a function of temperature for the Mn(Si_1−x_Sn_x_)_1.75_ samples.

The total thermal conductivities of Mn(Si_1−x_Sn_x_)_1.75_ are plotted in Figure 9a. All thermal conductivities decrease with increasing temperature. At 750 K, the 0.1 at% Sn-substituted sample exhibits the lowest thermal conductivity, approximately 2.19 W/mK. The obtained *k_total_* values were further deconstructed to delineate the contributions of lattice thermal conductivity (*k_L_*) and electron thermal conductivity (*k_e_*). A simplified single parabolic band model, with primary carrier scattering governed by acoustic phonons in the Boltzmann transport equation, was assumed. According to the Wiedemann–Franz law, *k_e_* is derived as *k_e_ = LσT.* The Lorenz number (*L*) typically ranges between approximately 1.5–2.44 × 10^−8^ *V*^2^ *K*^−2^ [36]. It has been observed that the conventional method of calculating *k_e_* underestimates its value due to the use of a constant *L*. To address this issue, an empirical formula (*L* = 1.5 + *exp*[−|*S*|/116]) has been proposed for a more accurate evaluation of the Lorenz factor in the calculation of *k_e_* [37,38]. Following this approach, the lattice thermal conductivity (*k_L_*) is determined using the equation *k_L_* = *k_total_* − *k_e_* = *k_total_* − *LσT*. For all Mn(Si_1−x_Sn_x_)_1.75_ samples, the calculated *k_L_* predominates in overall thermal conductivity when compared with *k_e_*. This predominance can be attributed to the reduced mean-free path of phonons due to Umklapp phonon processes, thereby elucidating the declining trend in lattice thermal conductivity observed between 300 K and 750 K. As shown in Figure 9b, the HMS sample doped with 0.1 at% Sn exhibits the lowest lattice thermal conductivity, 2.0 W/mK at 750 K. This value represents a notable 47.4% decrease compared to the undoped HMS sample. This result aligns with the observed peak shift towards lower angles in the XRD analysis, indicating that the presence of Sn induces lattice distortion and augments point defect scattering. Interestingly, the lattice thermal conductivity exhibits an upward trend with the Sn doping amount higher than 0.1 at%. As the Sn concentration increases up to 1.5 at%, a significant surge in lattice thermal conductivity was noted, surpassing that of the undoped HMS sample. This phenomenon can be attributed to the precipitation of metallic Sn within the matrix.

Figure 8d shows the dimensionless ZT values as a function of temperature for the Mn(Si_1−x_Sn_x_)_1.75_ samples. An appropriate Sn doping demonstrates effective suppression of thermal conductivity in HMS, exerting a relatively minor influence on the power factor. Specifically, the ZT value of Mn(Si_0.999_Sn_0.001_)_1.75_ exhibited a notable improvement to 0.31 at 750 K, a substantial enhancement over the undoped HMS. However, it is worth noting that ZT values exhibit a decline with excessive Sn doping amounts (x ≥ 0.005) due to increased thermal conductivity. These findings underscore that the primary role of Sn substitution in HMS is to reduce the lattice thermal conductivity rather than improving the power factor. In this study, the maximum power factor of Mn(Si_1−x_Sn_x_)_1.75_ remains lower than that of other HMS materials, such as (Al,Ge)-doped HMS [39]. Furthermore, the hole concentrations observed in Mn(Si_1−x_Sn_x_)_1.75_, 1.60–1.75 × 10^21^ cm^−3^, were lower than those of the optimized values in (Al,Ge)-doped HMS. This suggests an avenue for potential improvement in ZT within Sn-substituted HMS through a deliberate adjustment of doping parameters to optimize the carrier concentration or power factor in the Mn(Si_1−x_Sn_x_)_1.75_ system. This strategic tuning could potentially yield further enhancements in the ZT value. A comprehensive table of the TE parameters for all evaluated materials at 750 K is presented in Table 3.

## 4. Conclusions

In summary, this work synthesizes Mn(Si_1−x_Sn_x_)_1.75_ through arc-melting and SPS processes and systematically investigates the influences of Sn concentrations on the TE performance of HMS. Isoelectronic substitutions disrupt the arrangement of Si and Mn atoms, possibly leading to changes in the density of states (DOS) near the Fermi level. This change could arise from the possible formation of stacking faults within the HMS. As a result, it is likely that Sn substitution leads to changes in the electronic band structure and carrier density in Mn(Si_1−x_Sn_x_)_1.75_. On the other hand, the percentage of phase calculated from the XRD pattern with the RIR method reveal that the amount of HMS and MnSi are approaching the fixed value when x ≥ 0.001. It can be inferred that the precipitation of metallic Sn when x ≥ 0.005 predominantly affects the thermoelectric properties. As Sn precipitates from the HMS matrix, the large number of carriers causes the deterioration of thermoelectric properties. At first, the substitution of Si with Sn significantly reduces the lattice thermal conductivity of HMS by introducing point defect scattering when x = 0.001. In contrast to the undoped HMS, the lattice thermal conductivity diminishes to a minimum of 2.0 W/mK at 750 K for the Mn(Si_0.999_Sn_0.001_)_1.75_ sample, representing a substantial reduction of 47.4%. As a result of this suppressed lattice thermal conductivity due to Sn substitution, a noteworthy enhancement in ZT is achieved, reaching a value of approximately 0.31 at 750 K.

This work has demonstrated a significant increase in ZT for HMS, primarily attributed to the effective reduction in lattice thermal conductivity achieved through the incorporation of Sn. However, discrepancies between the actual and nominal compositions have been observed, attributed to the formation of secondary phases and clusters. The synthesis of pure HMS poses a challenge, largely due to the complexity of the Mn-Si phase diagram. Our future efforts will be directed towards improving the uniformity and homogeneity of HMS. Moreover, further improvement in ZT can be expected through a deliberate adjustment of co-doping parameters or employing nano-engineering techniques to optimize the power factor within the Sn-substituted HMS system.

## Figures and Tables

**Table 1:** Values of the lattice parameters and density for the sintered Mn(Si_1−x_Sn_x_)_1.75_ samples.

x	*a* (Å)	*c* (Å)	Density (g/cm^3^)
0	5.55	66.9	5.07
0.001	5.56	67.1	4.96
0.005	5.56	67.1	4.72
0.01	5.56	67.1	4.79
0.015	5.56	67.1	4.84

**Table 2:** Hole concentration and mobility of the sintered Mn(Si_1−x_Sn_x_)_1.75_ samples obtained from Hall measurements at room temperature.

x	Compositions of phase				Hole concentration (10^21^ cm^−3^)	Mobility (cm^2^ V^−1^ s^−1^)
	HMS	MnSi	Sn	SiO_2_		
0	92.8%	7.2%	N.D.	N.D.	1.55	1.47
0.001	94.7%	5.3%	N.D.	N.D.	1.60	1.37
0.005	94.9%	5.1%	N.D.	N.D.	1.65	1.35
0.01	94.2%	5.1%	0.7%	N.D.	1.72	1.31
0.015	93.9%	4.4%	1.7%	N.D.	1.75	1.29

N.D., none detected.

**Table 3:** Values of thermoelectric parameters for the studied materials determined at *T* = 750 K.

x	*σ* (10^4^ S/m)	*S* (μV/K)	*k_total_* (W/mK)	*k_L_* (W/mK)	ZT
0	1.896	213.9	4.0	3.8	0.16
0.001	1.627	234.8	2.2	2.0	0.31
0.005	1.786	222.3	2.7	2.5	0.24
0.01	2.189	213.4	3.5	3.2	0.22
0.015	2.211	211.7	9.1	8.9	0.08

## Data Availability

Data is contained within the article.
